# Understanding accreditation standards in general practice – a qualitative study

**DOI:** 10.1186/s12875-019-0910-2

**Published:** 2019-01-31

**Authors:** Tina Drud Due, Thorkil Thorsen, Marius Brostrøm Kousgaard

**Affiliations:** The Research Unit for General Practice and Section of General Practice, Department of Public Health, University of Copenhagen, Copenhagen, Denmark

**Keywords:** Accreditation, General practice, Primary care, Quality standards, Qualitative study

## Abstract

**Background:**

Accreditation is a widely adopted tool for quality control and quality improvement in health care, which has increasingly been employed for general practice. However, there is lack of knowledge of how accreditation is received and experienced by health professionals in general practice. This study explores how general practitioners (GPs) and their staff experienced the comprehensibility of accreditation standards and how they worked to increase their understanding of the standards. The study was conducted in Denmark where accreditation was mandatory in general practice from 2016 to 2018.

**Methods:**

The study consists of qualitative interviews with general practitioners and staff from 11 general practices that were strategically sampled among practices set to receive their survey visit in 2017. Participants were interviewed twice; once during the preparation phase and once after the survey visit. GPs and staff were interviewed separately. The interviews were analysed inductively using thematic analysis.

**Results:**

Understanding the requirements of the accreditation standards was a major challenge for the professionals when preparing for the accreditation survey visit. The participants attempted to increase their understanding of the standards in several ways including the use of regional support options and seeking out experts and colleagues. However, participants had mixed experiences with the various support options and many found the sense making work frustrating and time consuming.

**Conclusion:**

The results point to the importance of considering the level of specificity in accreditation standards and how to ensure an organisational set-up that can offer appropriate support to primary care clinics in terms of understanding what is required to meet the standards.

**Electronic supplementary material:**

The online version of this article (10.1186/s12875-019-0910-2) contains supplementary material, which is available to authorized users.

## Background

Internationally, accreditation is a widely adopted tool for quality control and quality improvement in health care [1]. In accreditation, an external institution assesses an organisation based on predefined quality standards and after a formal site visit by surveyors, the accreditation body decides whether to grant accreditation status to the organisation [2].

While accreditation originated in the hospital sector, accreditation has also become prevalent in general practice in several countries e.g. Australia, New Zealand, US, UK and Holland [3, 4]. In some countries, participation is mandatory, in others it is voluntary, but even when voluntary it is often linked to economic incentives [3, 5, 6].

In spite of the prevalence of accreditation and the considerable resources required for system-wide implementation, there is a lack of research on important aspects of accreditation [7]. While the number of studies on accreditation in hospitals has increased, there are few high-quality studies on the clinical effects of accreditation [7–9]. For general practice, the amount of research on accreditation is far more limited. A review of the literature by Hinchcliff et al. [7] only found 11 studies from general practice (compared to 79 from hospital settings).

Although some studies [6, 10, 11] have been published since that review, little is still known about the implementation and the consequences of accreditation at the clinic level in general practice. Such a lack of knowledge is problematic in terms of making more evidence-based decisions concerning the development, introduction, and implementation of accreditation systems [7].

In Denmark, a mandatory accreditation programme for general practice was implemented nationally from 2016 to 2018. Danish health care is mainly tax financed with free-of-charge access to general practice and public hospitals. GPs are private entrepreneurs mostly financed through the public health care reimbursement scheme and services are regulated by collective agreements between the Danish Regions and the Organisation of General Practitioners [12, 13]. Danish general practice is divided by 55% solo-practices and 45% partnership practices co-owned by two or more GPs (the latter covering 73% of the GPs) [14, 15].

Although the accreditation programme was primarily framed as a matter of quality improvement, a survey conducted before the initiation of the programme showed that almost half of the general practitioners (GPs) had negative attitudes towards accreditation, and that a large majority perceived accreditation to be an external control tool while about a third viewed it as both a control tool and a tool for quality improvement [16]. Further, half of the GPs were sceptical about the expected time consumption related to the accreditation process [16], a criticism that has also been levelled at accreditation programmes in other settings [1].

On this background – and as part of a larger research project on the implementation and effects of accreditation in general practice in Denmark [17] – we set out to do a qualitative study of how GPs and their staff experienced the accreditation process. Early in this project, it became clear that understanding the standards and their requirements was a major issue among the participants from general practice. Therefore, this paper investigates how GPs and their staff experienced the comprehensibility of the accreditation standards and how they worked to improve their understanding of the standards. A subsequent paper will address how the participants perceived the impact of accreditation on their practices.

## Methods

### Accreditation in Danish general practice

From 2010 to 2015, accreditation under the Danish Healthcare Quality Programme was mandatory in hospitals and then phased out. From 2016 to 2018, accreditation was mandatory in general practice. The Danish Institute for Quality and Accreditation in Healthcare (IKAS) was the agency in charge of conducting accreditation.

The set of accreditation standards for general practice was comprised of 16 standards representing four major themes: Quality and patient safety, Critical patient safety standards, Good continuity of care, and Management and organisation of the clinic (Table 1). These 16 standards were further broken down to 64 indicators.

**Table 1:** The 16 accreditation standards published in the handbook by IKAS

Name of standard	Focus areas
1. The professional quality	Use of diagnosis coding. Collection, analysis and use of clinical data for quality improvement.
2. Use of good clinical practice	Adherence to clinical guidelines particularly for diabetes and COPD. Special attention to vulnerable patients via a yearly plan for a selected group
3. Adverse events	Reporting, follow-up and process for learning in case of adverse events.
4. Patient evaluations	Completion of a patient evaluation and follow-up on the results.
5. Prevention of confusion of patient’s identity	Identification of patients principally by social security number and labelling of diagnostic material.
6. Prescription of medicine and renewal of prescriptions	Rational and safe medicine ordination and renewal of prescriptions. Participation in regional initiatives for correct medicine management. Annual assessment of patients’ list of medicine. Reporting of side effects.
7. Paraclinical tests	Execution of tests and handling of test materials. Quality control of equipment. Requisition and follow-up of paraclinical tests. Procedures for test results in case of GP’s absence. Procedures for missing tests results.
8. Emergency response and cardiac arrest	Handling of acute disease and cardiac arrest in the clinic. Participation in cardiopulmonary resuscitation course.
9. The patient health record, data safety and confidentiality	Content of patient health record conforms to current legislation. Journal audit performed and followed-up upon if needed. Safe storage, handling and destruction of sensitive personal data. Discretion and confidentiality for patients.
10. Availability	Accessibility in accordance with the collective agreement (e.g. telephone hours, opening hours and waiting time). Physical accessibility. Visitation of patients. Online practice declaration with relevant information.
11. Referral	Relevant and adequate content and handling of referrals.
12. Coordination of patient care	Coordination and continuity of patient trajectories in the clinic and in collaboration with other health care providers.
13. Acquisition, storage and disposal of clinical utensils and medicine/vaccines	Sufficient stuck of utensils, medicine and vaccines. Correct storage of medicine e.g. at the right temperature. Control of expiration dates. Correct disposal.
14. Hygiene	Cleaning of the clinic and inventory. Cleaning and storage of medical equipment. Correct hand hygiene. Management of infectious patients.
15. Management and operational activities	Ensuring good management via plans for quality improvement, division of responsibilities and tasks, quality control and development goals.
16. Hiring, introduction and competency development	Procedures for employing new staff with the right competences, for introducing new doctors and staff, for supervising staff and doctors in training and for ensuring on-going competency development.

The standards and indicators were developed by IKAS in collaboration with representatives from the Organisation of General Practitioners in Denmark, the Danish College of General Practitioners, Danish Regions, Danish Patients and the Danish Association of Practicing Medical Specialists. The standards had a dual purpose of control and improvement. Thus, the standards included some minimal requirements, but were also formulated in a relatively open way with the intention to stimulate reflection and quality improvement.

*“The standards include certain minimal requirements, but are also written to stimulate quality improvement. Thus, it would be a mistake to read the standards as one would read a regulation from the Danish Health Authority. Not everything in the standards is written to describe and delineate precisely what the client should do. Parts of the standards are intended to stimulate reflection on one’s own practice, and thereby inspire improvement activities.”* (IKAS’ webpage [18])

Practices were provided with a 52-page handbook. The book contained a description of the purpose and benefits of accreditation, the accreditation process, requirements for written procedures, the conduction of the survey visit, support functions (see below), definitions of key terminologies and 33 pages describing the 16 standards (1–3 pages each). The presentation of the standards were structured into Purpose, Content, Indicators and References. The specificity in the content of the standards varied, but they generally stated some overall requirements, and provided references to guidelines and other documents for the more detailed requirements. Most of the indicators simply stated that practices at the survey visit should be able to account for their work processes. Some standards required these descriptions to be textual while for other standards, verbal accounts of workflows were sufficient. As an example, an outline of the hygiene standards is provided in Table 2.

**Table 2:** An outline of the hygiene standard (Our excerpt and translation based on the handbook)

Purpose	
To prevent patients, relatives and staff from contracting infections in the clinic and from the reuse of equipment and materials.	
Standard content	
The standard presents four areas and describes the requirements for each area: cleaning of premises and fixtures, medical equipment and reusable materials, hand hygiene, infectious patients. For example, for medical equipment and reusable materials the requirements are: “The clinic has procedures for cleaning and storing medical equipment and reusable materials. Applied equipment must be cleaned in accordance with regulations issued by the supplier, The National Board of Health or other relevant actors. Sterile equipment and products should be stored properly to avoid contamination and exceeded expiry dates. Control of equipment for sterilization and cleaning of medical devices must be recorded in, for example, a logbook.”	
Indicators	
Six indicators are provided. For the area of medical equipment and reusable materials, the indicator is: “During interviews with the GP and practice staff they can explain procedures for cleaning and storing medical devices and reusable materials.”	
References	
For the whole standard eleven references are provided; the title and edition of ten different guidelines and the edition of the collective agreement for GPs.	

Throughout the accreditation period, information and materials to support the preparation work in the practices was provided on webpages from IKAS, DAK-E (The Quality Unit of General Practice), the five regional quality units, and Medibox (an online platform for continuing education). DAK-E also provided templates for uploading descriptions of work practices to IKAS prior to the visits. DAK-E and Medibox provided written examples of such descriptions for inspiration as to how practices could meet the standards. The regional quality units arranged information meetings and workshops, and offered support from accreditation consultants, medicine consultants, and data consultants.

The practices were notified a year before their scheduled survey visits, and each practice received 20.000 Danish kroner (approx. €2700) per GP in the practice (half of the amount was paid in advance and the rest when the practice had achieved accreditation).

### The design of the study

#### Study participants

The study consisted of qualitative interviews with GPs and staff (nurses and secretaries) from general practices (set to receive survey visits in 2017) in two Danish regions: The Capital Region and Region Zealand. The practices were strategically sampled [19] based on geography, practice type (solo/partnership) and a priori attitudes towards accreditation from a previous survey [12]. Practices were approached by e-mail and telephone. Practices who declined to participate in the study mainly stated the reasons as lack of time and energy. Originally, 12 practices were included in the study, but one practice was later excluded from the study, because their survey date was postponed. Information on the participants and their practices is presented in Table 3.

**Table 3:** Practices and participants in the study

Practice	Practice type	GPs and staff	1. interview participants	2. interview participants	A priori attitude to accreditation
1	Partnership	3 GPs, 1 nurse, 2 secretaries	2 GPs, 1 nurse, 1 secretary	1 GP, 1 nurse, 1 secretary	Negative
2	Solo	1 GP, 2 nurses	1 GP, 2 nurses	1 GP, 2 nurses	Positive
3	Partnership	3 GPs, 2 nurses, 3 secretaries	3 GPs, 2 nurses, 1 secretary	3 GPs, 2 nurses, 1 secretary	Negative
4	Solo	1 GP, 1 biomedical laboratory scientist	1 GP, 1 biomedical laboratory scientist	1 GP, 1 biomedical laboratory scientist	Positive
5	Solo	1 GP, 1 secretary	1 GP, 1 secretary	1 GP, 1 secretary	N.A.
6	Partnership	3 GPs, 3 nurses, 1 secretary	3 GPs, 2 nurses, 1 secretary	3 GPs, 2 nurses, 1 secretary	Positive
7	Solo	1 GP, 1 nurse	1 GP	1 GP	Negative
8	Partnership	2 GPs, 2 nurses	2 GPs, 2 nurses	2 GPs, 2 nurses	Negative
9^a^	Partnership	2 GPs, 1 secretary	2 GPs	-	Positive
10	Solo	1 GP, 1 nurse	1 GP, 1 nurse	1 GP, 1 nurse	Negative
11	Solo	1 GP, 1 nurse	1 GP, 1 nurse	1 GP, 1 nurse	Positive
12	Partnership	3 GPs, 2 nurses, 2 secretaries	3 GPs, 2 nurses	3 GPs, 2 nurses	Negative^b^ Positive

^a^ Survey postponed, practice excluded from the study^b^ Two different GPs had answered the questionnaire

#### Qualitative interviews

We initially conducted pilot interviews in two practices that had completed the accreditation process, to explore planned themes for the interview guides and identify potentially new ones. We had an explorative approach in both data collection and analysis. However, in order to make sure that we covered potentially important dimensions of an implementation process, the interview guides were also inspired by Normalisation Process Theory (NPT). NPT is a middle range theory of implementation focusing on how actors understand a new intervention and are able to differentiate it from existing practice (coherence), engages with the intervention individually and collectively (cognitive participation), transform it into practice under specific organisational conditions (collective action), and how they evaluate it (reflexive monitoring) [20].

Representatives from the included practices were interviewed in the practices twice by the first and last author (TDD and MBK). The first interview was conducted 3–8 months before their survey visit as the practice was preparing for the visit; the second interview was conducted 2–7 month after the survey visit. GPs and staff were interviewed separately, and each interview lasted about one hour. The interviews were semi-structured and the interview guides were adjusted a few times during the process to incorporate perspectives and focus areas uncovered in previous interviews. Table 4 displays the overall themes related to the accreditation process from the two interview guides, and a translated edition of the interview guides is accessible in the paper’s Additional file 1. We emphasised to the participants that we were researchers with no affiliation to IKAS, the Regions or other stakeholders, and that we did not have vested interests in specific study outcomes. Furthermore, all participants were promised anonymity and confidentiality and we emphasised that no identifiable information would be given to neither IKAS nor other involved partners.

**Table 4:** Themes in the interview guides

1.	Thoughts and attitudes regarding the accreditation programme and the specific standards.
2.	Expectations about the impact of accreditation on quality.
3.	Factors promoting or inhibiting working with the standards
4.	Understanding what it takes to comply with the standards.
5.	Ways of improving understanding.
6.	The accreditation work process and division of tasks.
7.	Participation in regional support activities.
8.	Use of examples of procedures.
9.	Sharing experiences with colleagues.
10.	Writing new procedures and expectations to their use.
11.	Working with the accreditation standards vs. working with other quality improvement initiatives.
12.	Time consumption.
13.	Expectations about the survey visit.

### Analysis

All interviews were audio recorded, transcribed verbatim, and inductively analysed with thematic analysis based on the approach of Braun and Clarke [21]. We used the software program NVivo, and all three authors participated in the coding process and analysis. First, we read and summarised the interviews to get an overview of potential themes, and we discussed and agreed upon a coding structure. Using this, we coded two interviews simultaneously, compared our coding, and discussed and refined the coding structure. Our coding-tree is illustrated in the paper’s Additional file 2. We then coded the remaining interviews and composed new comprehensive summaries for each practice based on coded extracts from all interviews performed in the practice to get an overview of the codes and themes in each practice. Hereby, we could outline the process in each practice, and identify divergence and connections between extracts from different codes. We then compared themes and coded extracts both within and across practices, and wrote a coherent narrative of each theme. In case of questions or puzzlement, individual interviews were re-read.

Part of our pre-conceptions was the inspiration from NPT, which gave rise to an attention to practices’ understanding of the requirements, sense making work and preparation process as well as potential challenges in these areas. Furthermore, we were aware of an accreditation scepticism among GPs in the public discourse, of diverse a priori attitudes identified in the previous survey study, and of challenges identified in studies of accreditation internationally in general practice and hospitals. We paid attention to this in the sampling procedure, and in the interviews and analysis, we were conscious of and challenged our pre-conceptions. As social scientists, we had an outsider’s view and had no vested interests in the outcome of the study.

## Results

The analysis showed that understanding the requirements of the accreditation standards was a widespread challenge among the practices. In the following, we elaborate on the practices’ perceptions of the comprehensibility of the standards, how uncertainty generated problems when describing local work practices, and how the practices sought to increase their understanding. Finally, we outline some important variations between the practices in their aspirations, approaches and time consumption.

### Perceptions of the standards’ comprehensibility

At the beginning of the preparation process, almost all practices had experienced some degree of uncertainty concerning their understanding of the accreditation standards. Some participants described problems with the readability of the standards due to the style of language in the standard book (and some of the referenced documents) which they viewed as being too distant from their daily practice, too ‘theoretical’ and ‘legal-like’. For some, the language style made understanding seem even more difficult in the beginning than it was later perceived to be. For others it did not get much clearer during the process.

Further, some participants had found it difficult for their overview and understanding that the standards contained references to several different concepts, agreements, guidelines and regulations. Thus, many standards were much more extensive than they appeared at first sight. As a GP described it:

*“It’s like reading Buddhist texts. They can be very short and yet contain an entire universe. I mean, there are several things where you have to ask yourself: what do they really mean by this?”* (Practice 7, GP)

Thus, in order to get familiar with the actual content and possible implications of the standards, the participants had to read several related documents. In some cases, the participants perceived discrepancies between these documents (e.g. hygiene and work environmental guidelines) which created further confusion.

For the majority of the participants, however, the most significant issue of understanding was that the standards were perceived as too diffuse and unspecific making it difficult for the participants to pin-point exactly what behaviour was expected of them, and how this differed from their usual practice. While a few of the participants saw benefits of not having all the answers from the beginning in the form of very detailed standards, because they considered the discussions in the practice to be the most important part of the process, several participants wished that the standards had been more explicitly and comprehensively described – like a recipe in a cookbook. They perceived a contradiction between the control dimension of accreditation on the one hand and the low level of details in the requirements on the other:

*“We had to reinvent the wheel – It would have been much easier to go through this accreditation process if things had been defined in advance, e.g. “the refrigerator has to have that temperature… the hygiene has to be like this and this” […] if it had been like that we would have had no problems. The greatest hurdle was to find out what we had to comply with”* (Practice 6, GP)

Even participants who generally found the requirements to be clearly described pointed to some areas that lacked clarity. There were also examples of practices that believed that they conformed to all of the requirements, but nevertheless received remarks at the survey visits.

Some standards were particularly challenging to understand in terms of the exact requirements, including: Hygiene; Acquisition, storage, and disposal of clinical utensils and medicine/vaccines; Paraclinical tests; Prevention of confusion of identities; and requirements about notation of informed consent (see Table 5 for examples).

**Table 5:** Examples of experienced uncertainties

Standard	Areas of uncertainties
Hygiene	• Correct sterilisation procedure for certain instruments. • Discrepancy in hygiene and work environmental guidelines regarding the use of chlorine-containing product. • Frequency of testing of autoclave. • Frequency and type of cleaning of the clinic. Daily cleaning was required, but uncertainties about what that entailed. • Frequency and type of cleaning of certain instruments like blood pressure monitor and ear thermometers and warming cabinet. • When documentation in a logbook is required.
Acquisition, storage, and disposal of clinical utensils and medicine/vaccines	• For measurement of refrigerator temperature uncertainty about which thermometer to use, the frequency of measurement, and the required documentation. • Required systematisation, frequency and documentation for control of expiration date of medicine.
Paraclinical tests	• The scope of GP’s responsibility for following up on paraclinical tests in relation to: a) ensuring that patients are informed about test results b) checking that paraclinical examinations referred to external providers have actually been carried out, and contacting the patient if he/she has not attended the examination.
Prevention of confusion of identities	• Whether patients always have to be identified by social security number or if/when facial recognition is sufficient. • If cultivations labelled with patient’s full social security number are in contradiction with requirements about patient discretion (no visible social security numbers in the practice).
The patient health record	• How informed consent must be ensured - whether the GP has to ask directly and how it is to be noted in the patient record.

For some practices, the comprehensibility of the standards was improved considerably during the process of working with the standards (see the section below on ‘Working to improve understanding’). Others were still frustrated by uncertainty far into the process, even right up to the survey visit:

*“Right now, we have no idea; have we been doing far too much, or is it completely wrong what we have been doing? Could we have managed it with a fraction of the work, or will we flunk? I mean, what level are we at?”* (Practice 1, GP)

### Uncertainty and the description of local work practices

As mentioned above, many standards required that the practices could present the surveyors with documents describing aspects of their work. Some practices already had such descriptions covering parts of their work whereas others had few.

While converting existing work practices and verbal instructions into text could be difficult in itself (e.g. finding the right words and allowing for the contingencies of patient care), this challenge was exacerbated by the problems of understanding the requirements of the standards. Thus, several practices found the writing process demanding and time consuming, particularly due to uncertainties about requirements:

*“Because you become uncertain it takes extra time and you don’t know exactly what they expect. Does every single little thing have to described in detail?”* (Practice 1, Secretary)

In one practice where the professionals were sure that they already acted in accordance with the standards, they had nevertheless spent time worrying and discussing how to describe their procedures in the right way, fearing the consequences of a wrong phrasing:

*“So we have spent a lot of time discussing and writing down and [asking ourselves:] ‘is this written in the right way? Will they use this against us?’ We have used an enormous amount of energy on that”*(Practice 6, Nurse)

As it also indicated by this quote, uncertainties about the standards’ requirements were often associated with uncertainties about the nature of the survey visits and the zealousness of the surveyors in their assessments of compliance with the standards. This uncertainty was the reason why some practices had formulated their procedures in a more complicated and more official manner than if the documents had been for internal use only.

In some practices describing local routines in writing became easier during the process as the participants gained more experience with the format. Also, a few practices did not experience any difficulties in writing down their procedures either because they found the requirements clear and/or because they did not worry too much about the exact phrasing:

*“I think that if you just write down how you do things, then that must be sufficient”* (Practice 8, GP)

### Working to increase understanding

Apart from (re-)reading the standards (and the related documents) and engaging in internal discussions in the practice, the participants had sought out information and clarification from different sources in order to increase their understanding of the requirements and how to conform to them.

#### Seeking understanding through regional support arrangements

All but a few practices had attended information meetings (or workshops) provided by the regional quality units and most of them described some benefits from participating. At the meetings, they had received practical information (including where to find examples of how to describe clinical and administrative work procedures) as well as input from other practices. Some participants experienced that the meetings had reassured them of being on the right track and had served to demystify accreditation, because they had learned that the requirements were less comprehensive than they had believed. However, others did not find much value in these meetings, because the information was not concrete enough, or because the timing of the meeting was off in relation to their own process of working with the standards (i.e. they had not yet begun the work or they were much further ahead than the rest of the participants at the meeting). Further, some experienced that the presenters at the meetings were not able to answer their questions about specific standards.

The majority of the practices in the study had not contacted the regional accreditation consultants for support – in most cases because they were not aware of this option. One practice that were aware of this option, had chosen not to use it, because they did not expect that the regional consultants would be able to answer their questions (since they did not represent IKAS) and because they did not know any of the regional consultants. Contrary, another practice had called the regional consultant frequently. They experienced this service as vital, and viewed the consultant as their ‘guru’ and credible hotline, who had provided examples of written procedures and answered their questions many times when they had been in doubt, saving them a vast amount of time on discussions and looking for answers. Prior to contacting the consultant, they had experienced working with the standards as confusing and time consuming. A few practices had also requested visits from the regional data consultant concerning the standard on data security and they found this to be beneficial since the consultants could provide specific technical advice.

#### Seeking understanding through examples

The examples of written procedures provided online by DAK-E had been used profoundly by almost all of the practices in the process of describing their own work routines. The professionals generally appreciated these examples, which had increased their understanding of how to respond to the standards. Hence, when formulating local written procedures, the examples had provided direction in respect to the level of detail, specific phrasings, and structure of the document. Some practices mainly used the examples as inspiration, others talked about them as a template, and a few had copied them directly when the descriptions corresponded with their own procedures.

However, some participants from solo practices reckoned that some of the examples were more applicable to larger practices and hence too extensive for them. Others found the examples to be lacking in detail and would have preferred them to be more comprehensive, directly applicable, approved by IKAS, and divided into practice types so that the clinics would not have to spend as much energy on describing their procedures in writing:

*“[the process] of writing down [your procedures] is difficult and demanding for many [practitioners] […] that process has created negativity, which was completely unnecessary in my view […] If more [examples of] written procedures had been available, which the clinics could then subscribe to or adjust to fit their own activities, that would have been a great help” *(Practice 11, GP)

Finally, some participants had come across examples from several sources (DAK-E; colleagues, and Medibox) and experienced that the content and extent of these examples varied. This provided them with different inspirations, but the variations could also lead to uncertainty about the correct way of doing things.

A few practices had not used the examples, because they had not been aware of their existence, or because the practices had started their preparation process before the examples had been available. The participants from these practices were frustrated that they had not been provided with the examples from the beginning since they had needed this kind of support.

#### Seeking understanding through other experts or colleagues

The practices also sought out information from other formal and informal sources than those established in relation to the accreditation programme. Hence, several practices had contacted the Serum Institute (‘Statens Serum Institut’) or other experts, seeking clarification on hygiene requirements, and one practice had arranged a visit from a hygiene consultant from their equipment provider, to assess what they needed to change in order to adhere to the requirements. Others mentioned they would have liked a visit from a hygiene nurse.

Practices also ‘googled’ for answers and sought support from their colleagues – most commonly through informal talks at meetings and in Facebook groups for GPs or practice nurses. A few participants had seen documents produced by other practices, but generally, this kind of communication was not very structured. For the professionals, the advantages of informal collegial support, especially in the Facebook groups, were that they learned how other practices had interpreted the standards and that they became aware of things they had not considered in particular standards. They also heard how the survey visits had been conducted in other practices, and what topics the surveyors had paid most attention to. At times, such information had a calming effect concerning the sense making work and what to expect from the survey visit. However, at other times participants experienced increased uncertainty from hearing about the different ways that other practices had interpreted and implemented the standards, and about the other practices’ very different experiences with the survey visit and subsequent assessment:

*“There has been a lot of rumours about what things the surveyors attached importance to and that was very different things… so when you heard stuff like that you were like ‘oh my God no, then we also have to do this and that’ and then you start to do a lot of new things”* (Practice 1, GP)

The participants described that the questions both they and others asked on Facebook were often very concrete and aimed at clarifying the proper interpretation of the standards. This was by some described as inexpedient, since they believed such questions should have been addressed directly to the accreditation institution. However, they did not experience this as an option, and since they felt that they were left to themselves, they looked to their colleagues for advice and discussion.

*“In the Facebook group, there are a lot of those very specific questions like ‘how do you do this? What about that centrifuge? and what about that and that?’ So it’s very specific questions that are raised in that group […] but that is not expedient. Where else could they ask those questions, because you can’t write to IKAS [and ask]: ‘how many times a week is the centrifuge to be..’ or something like that, because they won’t answer that. ‘You have to find that out for yourself”* (Practice 6, Nurse)

### Variations in aspirations, approaches and time consumption

The efforts related to understanding the standards and formally describing their work procedures were experienced as very time consuming in most of the practices, including some of those that were positive about accreditation and some of those that were used to working with quality improvement and describing their procedures:

*“I think we agree here in this house that it makes really, really good sense to do all these things, but the road to getting there is simply so ridiculous”* (Practice 6, Nurse)

And

*“Even for us – and we had not expected that since we believe that we have a good hold of things in our practice. Even for us it was very, very time consuming because… ‘is this good enough and what do they really mean, and how were we supposed to put it into writing it?’ and things like that”* (Practice 1, GP)

In most practices the participants believed that they could have spent less time and achieved the same had the requirements been more specific and had there been more detailed examples of written procedures for all standards.

However, the challenges, concerns, and time consumption related to understanding the standards and describing their local work routines varied between the practices depending on the participants’ level of aspirations, their expectations to the survey visits, and their mental approach to preparing for the visit. This variation can be illustrated by the two very different cases of practice 5 and practice 6.

In practice 6, all GPs and most staff had been deeply involved in the preparation process and the level of aspiration was very high in the sense that they wanted to be absolutely sure that they got accredited in the first attempt without any remarks even if this meant that they were likely over-implementing. However, wanting to make sure to be accredited coupled with uncertainties about several of the standards led the participants from this practice to worry exceedingly and engage in many detailed discussions about how exactly to understand and conform to the standards. They also went through all their procedures several times up to the survey to ensure that everything was in place (for example by performing small test surveys and quizzes on the standards).

Contrary, the GP in practice 5 had taken a much more relaxed approach to accreditation, comparing his approach to accreditation with the idea of having his car for inspection. Therefore, although the practice had made some changes prior to the survey, the GP mainly waited for the survey visit to make clear what changes had to be made in order to get accredited. Thus, the GP did not worry whether everything was correct, and by delegating much of the work of making sense of the standards to the surveyors, the GP had spent much less time preparing for accreditation than the other GPs in the study. However, this was also the only practice that received remarks to a degree that an additional survey was required.

For the rest of the GP-participants this relaxed approach was not an option since they aspired ‘to pass’ in their first attempt (the first survey visit). For some this was a question of pride and reputation and/or of being perfectionist in nature:

*“I have a high sense of honor… I refuse to have it said that it did not work out [in my clinic]. [When] I think of my colleague [in another practice] who did not achieve accreditation; that must be so embarrassing! [Laughing] […] I think, it is an admission of failure not to be able to conform to a set of given standards”* (Practice 11, GP)

For others it was more about not having to spend time on an additional survey visit (which required the practice to close for most of the day):

*“The ambition is to pass the first time. And to spent as little time as possible”* (Practice 8, GP)

Some of these practices also expressed a tendency of over-implementation due to uncertainties about the requirements, and after the survey several participants felt that some of their preparation work had not been required to receive accreditation status.

## Discussion

This study found that understanding what was required to comply with accreditation standards was perceived to be a substantial challenge by the professionals from several practices in the process leading up to the survey visit. In order to improve their understanding of the standards, the participants engaged in various kinds of sense making work, including: Finding and reading related guidelines and regulations; internal discussions of the standards and how to meet their requirements; attending regional information meetings and workshops; locating formal examples of standards; and seeking advice from experts or colleagues in other practices. These efforts were generally perceived as time consuming and the practices had mixed experiences with the various support options. There were marked variations in the participants’ aspirations and approaches concerning the accreditation process and they had different thresholds concerning the level of understanding that they deemed to be satisfactory.

Within implementation theory, the attempts of organisational actors to understand how the ideas, recommendations or requirements associated with new interventions differ from existing work practices have been termed *differentiation* [20]. Our results highlight the important role of such differentiation in the local implementation of a mandatory accreditation programme in general practice and this aligns with literature on clinical guidelines, which has found it to be critical for implementation that the guidelines are easy to understand [22]. In order to facilitate implementation this literature has recommended the involvement of professionals in the design process and in the testing of guidelines in small scale prior to implementation [22]. Similar recommendations would seem to apply for the development of accreditation standards. In Denmark, representatives from general practice did take part in the design of the accreditation standards and a first version of the standard set was piloted in 2012, four years before the launch of the national accreditation programme. Interestingly, the evaluation report from this pilot project pointed to problems with unclear language and difficulties with understanding the required level of implementation. Therefore, it was recommended that a subsequent national accreditation programme should employ more specific standards, and offer a high degree of implementation support including instruction materials with examples [23]. Yet, the present study has identified the same kind of problems, even in some practices that were positive about accreditation and some that were experienced working with quality improvement, and this explicates that ensuring a good understanding of the requirements among the health practitioners is a profound challenge.

In terms of support, the accreditation agency, the five regions and the DAK-E provided various kinds of support during the process, but the question is whether this was sufficient, and whether information about these support options was efficiently disseminated. Also, the fact that three different actors provided information and support might have decreased the practices’ overview of these options. Further, though all practices appreciated the provided examples of how to meet the requirements when describing local work routines, several participants would have preferred the examples to be even more detailed and directly applicable.

The general challenge of understanding the requirements seem to imply that the standards should have been less open to interpretation and more specific. However, basing an accreditation programme on very specific standards raises a number of issues to be considered: First, the standards have to accommodate a heterogeneous practice sector where one size does not always fit all [11], and hence ‘too’ rigid standards may not be implemented in daily practice. Second, more specific standards are more likely to require updating thereby adding to the work of the accreditation agency. Third, in principle, all standards (like guidelines and rules) are always open to – and require – some kind of context-based interpretation [24, 25]**,** and this sets limitations on the obtainable level of common and immediate understanding across different sites. Finally, as mentioned in the Methods section, a central idea behind most of the standards was not to prescribe the exact activities to be implemented but to inspire practices to engage in improvement activities. From this perspective, shifting the balance towards more specific standards can be seen as a step towards increased regulation, which may be counterproductive to the objective of quality improvement. On the other hand, the framing of accreditation as a matter of improvement rather than control seemed difficult to communicate to the health professionals for whom the mandatory framework of standards, survey visit, and final verdict on accreditation status signalled regulation. These different objectives of accreditation (regulatory compliance to minimal standards vs. continuous quality improvement) have also been present in accreditation models in Australia, where stakeholders currently discuss if and how they can be fruitfully combined [26].

The above considerations lead us to conclude that it might be necessary to keep some standards defined in more general terms, but then linking to various examples (approved by the accreditation agency) that illustrate how each standard can be implemented in different circumstances as well as a list of points in need of attention. This would leave room for discussions on local tailoring. In other areas where the accreditation agency applies more specific and inflexible assessment criteria, these criteria should preferably be fully transparent in the standard. These reflections align with the observation by Timmermans & Epstein that “[t]he trick in standardization appears to be to find a balance between flexibility and rigidity and to trust users with the right amount of agency to keep a standard sufficiently uniform for the task at hand” [25].

### Strengths and limitations

Interviewing practices twice ensured more detailed descriptions of their processes. The first interview offered insights into the process midway and better recollection of initial activities and experiences; whereas the second interview offered insights into the remaining process and potential changed perceptions, and enabled follow-up questions. With informant triangulation, interviewing both GPs and staff and doing it separately, we obtained a more detailed picture of each practice as a whole plus shared and divergent views between these groups.

We believe to have reached reasonable data saturation, but given that the 11 clinics included in this study constitute a small sample compared to the approximately 1800 general practices in Denmark, it is difficult to say to what extend the results apply to the rest of the practice population. However, we believe that this qualitative study did identify some widespread issues, and that the strategic sampling ensured that the articulated experiences were not merely connected to certain types of practices or to professionals with similar attitudes to accreditation. Further, data from IKAS has shown that the standards, which the practices in our study had most difficulties in understanding (Hygiene; Paraclinical tests; Prevention of confusion of patient’s identity) were also those most frequently causing remarks at the survey visits [27]. Regarding the transferability of the findings in an international perspective, the study focused on the first accreditation programme in general practice in Denmark. In countries where accreditation programmes have been in operation for several years in general practice, the issues and concerns related to understanding may not be as prominent, since many questions related to the assessment criteria and the survey visit will have been clarified. Therefore, the results may be more applicable to primary care settings where accreditation is introduced for the first time. Still, mixed experiences with support arrangements and some levels of confusion concerning the assessment process have also been reported in more established programmes [6].

## Conclusion

The study found that understanding the requirements of the accreditation standards was experienced as a challenge among the professionals causing varying levels of frustration and spurring various efforts aimed at increased understanding. This sense making work was often seen as too time consuming and as something that could have been minimized if the standards had been more explicit. These results underline the importance of ensuring clarity in accreditation requirements and of providing easily available support (preferably with one access point) that can accommodate the varying needs of the participating practices. At the same time, the results point to the importance of carefully considering the balance between flexibility and specificity in accreditation programmes as well as other types of governing programmes relying on quality standards.

## Additional files

Additional file 1: Interview guides. A translated edition of the interview guides for both the first and second interviews with the themes concerning practices’ attitudes and accreditation processes. (DOCX 20 kb)

Additional file 2: Coding tree. A visual representation of our coding tree. (DOCX 65 kb)

## Abbreviations

DAK-E: The Quality Unit of General Practice
GP: General practitioner
IKAS: Danish Institute for Quality and Accreditation in Healthcare
Medibox: Online platform for continuing education
NPT: Normalisation Process Theory
