# Supplementary material for: Understanding accreditation standards in general practice – a qualitative study
Source: BMC Fam Pract. 2019 Jan 31;20:23. doi: 10.1186/s12875-019-0910-2 (PMC6354356; doi:10.1186/s12875-019-0910-2)
Supplement: Supplementary file 1 — Interview guides. A translated edition of the interview guides for both the first and second interviews with the themes concerning practices’ attitudes and accreditation processes. (DOCX 20 kb) [file 12875_2019_910_MOESM1_ESM.docx]

# Additional file 1: Interview guides

| **First interview**  Given the semi structured interview form the content, focus and order of the interview was flexible and dependent on how far along each clinic was in their process and their responses to the questions. | |
| --- | --- |
| **Introduction** | - Information about the interviewer, the research project, confidentiality and anonymity. - Information about the structure of the interview. - Questions about the clinic’s organisation, staff and those present at the interview. |
| **Coherence** | 1. What did you think when you first heard that general practice was to be accredited?  - Follow up: Thoughts after having read the standards and started working with them.  1. What do you think those who have decided that general practice should be accredited consider as the purpose? 2. What do you see as the purpose of accreditation?  - Follow up: Thoughts concerning the discussion on control vs. quality development]  1. Do you find that you in the clinic agree on your views on accreditation and how to work with it? 2. Do you have a clear understanding of the individual standards?  - Follow up:   - Are some standards more difficult to understand than others?   - Do you have a clear understanding of what is needed before you can be accredited? [in general and in the individual standards]   - Does the clinic have a common understanding of the individual standards and what it takes to live up to them?  1. Do the standards indicate a different way of doing things than usual? 2. Assuming that you live up to all the standards when you finish this process - how do you think it will affect the quality of your services in this clinic?  - Follow up: Why / how / why not?  1. What is your assessment of the professional relevance of the standards?  - Follow up:   - The relevance to general practice as a whole   - The relevance for your clinic   - The relevance of the indicators in the individual standards  1. What are your thoughts about the basis of the standards?  - Follow up: In relation to professional knowledge, science, evidence-based  1. How do you see accreditation as a tool for quality improvement? 2. How does the rest of the practice [GP and staff not present in this interview] perceive accreditation? |
| **Cognitive Participation** | 1. How would you describe your approach to working with this accreditation task?  - Follow up:   - To be accredited with the least possible effort, seeing it was an occasion to develop your practice, checking what you do is consistent with the standards/ is good enough.   - In the beginning and now that you have worked with the standards  1. Up until now how have you been working with the standards?  - Follow up:   - Who has taking the initiative   - Meetings held and their content   - Division of tasks – the role of the staff  1. How much time have you spent understanding and working with the standards? 2. Have you participated in the regions support activities [information meeting, workshops]  - Follow up:   - Content and usefulness   - Reason for not participating |
| **Collective action** | 1. What tasks have each of you carried out as part of the work of meeting the standards?  - Follow up: Have you read guidelines and disease management programmes in connection with working with the standards (e.g. COPD and diabetes)?  1. How has it been working with the standards?  - Follow up:   - Which standards are (have been) easiest to live up to / to implement and why?   - Which standards are (have been) the most difficult to live up to and why? [In case the informants do not come up with anyone, ask about standards others have mentioned as problematic (hygiene and ensuring test results)]   - How have you dealt with the problems?  1. What has eased and impeded working with the standards – now and in the future?  - Follow up: Are there things in their organisation that influence this (e.g. solo vs partnership practice)?  1. Have you used the DAK-E system (examples and uploading documents to IKAS)?  - Follow up: For what and how has it worked?  1. Have you used your colleagues while working with the standards?  - Follow up: In what way and experience of benefits and limitations  1. Have you been lacking any competences while working with the standards (IT, project management etc.)? 2. Have you produced written documents of your procedures as part of the accreditation process?  - Follow up:   - If yes which ones   - What does it mean to have the procedures written down  1. Have you performed the required journal audit, the patient evaluation, chosen a vulnerable patient group?  - Follow up:   - Thoughts about these requirements.  1. Are there things you already do differently than before due to having worked with the standards [or is it rather existing practices that are now written down, or former written procedures revised]?  - Follow-up: The extend they use or expect to use the new procedures  1. Are there things you have chosen not to live up to? 2. Did you have sufficient time to work with the standards and the related activities ensuring you live up to them?  - Follow up: What are your thoughts about the financial reimbursement in relation to accreditation  1. How has working with the standards been compatible with other change and quality improvement processes in the clinic? |
| **The survey visit** | 1. How does it influence working with the standards that you are visited by a surveyor who assesses if you live up to them? 2. What thoughts do you have about the survey visit? 3. How will you prepare for the visit? 4. What are your thoughts about the surveyor being a colleague [only the GP and in some cases the staff]? 5. Do you expect to be accredited?  - Follow up: What would it mean to you, if you were not accredited? |
| **Closing** | 1. Are there something you want to add, something important we have not talked about concerning your process or the specific standards? |
|  | |
| **Second interview**  Depending on the clinic’s status at the time of the first interview, some questions from the first interview guide were used at the second interview. Further, there were follow-up questions specific to each practice dependent of their responses at the first interviews. Again given the semi structured interview form the content of the interview was flexible and dependent on their responses. | |
|  |  |
| **Introduction** | - Information about confidentiality and anonymity - Information about the content and structure of the interview |
| **Process after first interview** | 1. What was your process between our first interview and your survey?   Follow up questions specific to each practice about their process before the survey visit based on their first interview.   1. …. 2. …. 3. ….     Follow up questions to all practices about the process before the survey based on the first interviews.   1. To what degree was it uncertain what you had to live up to and to write down?  - Follow up: In which areas could something more specific be beneficial e.g. a checklist?  1. What material did you read while working with the standards?  - Follow up: Where else did you find information?  1. Last time you told us that you had used the written examples of the procedures can you elaborate on how you used them more concretely? 2. One practice in this study had a visit from a regional consultant and called her several times when they were in doubt – have you heard about this option, considered using it or at other times contacted the region or IKAS with questions?  - Follow up: Is it due to lack of knowledge hereof, that it was not needed or something else?  1. Do you expect to use the documents you have made describing your local procedures yourself or are they mainly used to live up to the requirements?  - Follow up: Are they written differently, because they need to be used for accreditation and uploaded to IKAS (formulations, structure etc.)?  1. Did you feel ready for the survey visit? 2. With the knowledge you have now (after the survey) is there something you would have done differently in the process before the survey? |
| **Questions in the guide not relevant for this paper** | There were several question within the following themes:   - Their description and experience with the survey visit - The time after the survey - How they perceive the impact of accreditation on their practice - Their thoughts about accreditation now and in case of future accreditation |
