# Supplementary material for: Understanding accreditation standards in general practice – a qualitative study
Source: BMC Fam Pract. 2019 Jan 31;20:23. doi: 10.1186/s12875-019-0910-2 (PMC6354356; doi:10.1186/s12875-019-0910-2)
Supplement: Supplementary file 2 — Coding tree. A visual representation of our coding tree. (DOCX 65 kb) [file 12875_2019_910_MOESM2_ESM.docx]

# **Additional file 2: Coding tree**
